# Precision psychiatry with immunological and cognitive biomarkers: a multi-domain prediction for the diagnosis of bipolar disorder or schizophrenia using machine learning

**DOI:** 10.1038/s41398-020-0836-4

**Published:** 2020-05-24

**Authors:** Brisa S. Fernandes, Chandan Karmakar, Ryad Tamouza, Truyen Tran, John Yearwood, Nora Hamdani, Hakim Laouamri, Jean-Romain Richard, Robert Yolken, Michael Berk, Svetha Venkatesh, Marion Leboyer

**Affiliations:** 1 Center of Excellence on Mood Disorders, Department of Psychiatry and Behavioral Sciences, McGovern Medical School, The University of Texas Health Science Center at Houston (UTHealth), Houston, TX USA; 2 IMPACT – the Institute for Mental and Physical Health and Clinical Translation, School of Medicine, Barwon Health, Deakin University, Geelong, Australia; 3 School of Information Technology, Deakin University, Geelong, Australia; 4 Applied Artificial Intelligence Institute (A2I2), Deakin University, Geelong, Australia; 5 AP-HP, Université Paris Est Créteil, Department of Psychiatry and Addictology, Mondor University Hospital, DMU IMPACT, Translational Neuro-Psychiatry laboratory, INSERM U955, Créteil, France; 6 Fondation FondaMental, Créteil, France; 7 Stanley Neurovirology Laboratory, Johns Hopkins School of Medicine, Baltimore, US; 8 Florey Institute for Neuroscience and Mental Health, Department of Psychiatry and Orygen, The National Centre of Excellence in Youth Mental Health, University of Melbourne, Parkville, Australia

**Keywords:** Diagnostic markers, Molecular neuroscience

## Abstract

Precision psychiatry is attracting increasing attention lately as a recognized priority. One of the goals of precision psychiatry is to develop tools capable of aiding a clinically informed psychiatric diagnosis objectively. Cognitive, inflammatory and immunological factors are altered in both bipolar disorder (BD) and schizophrenia (SZ), however, most of these alterations do not respect diagnostic boundaries from a phenomenological perspective and possess great variability in different individuals with the same phenotypic diagnosis and, consequently, none so far has proven to have the ability of reliably aiding in the differential diagnosis of BD and SZ. We developed a probabilistic multi-domain data integration model consisting of immune and inflammatory biomarkers in peripheral blood and cognitive biomarkers using machine learning to predict diagnosis of BD and SZ. A total of 416 participants, being 323, 372, and 279 subjects for blood, cognition and combined biomarkers analysis, respectively. Our multi-domain model performances for the BD vs. control (sensitivity 80% and specificity 71%) and for the SZ vs. control (sensitivity 84% and specificity 81%) pairs were high in general, however, our multi-domain model had only moderate performance for the differential diagnosis of BD and SZ (sensitivity 71% and specificity 73%). In conclusion, our results show that the diagnosis of BD and of SZ, and that the differential diagnosis of BD and SZ can be predicted with possible clinical utility by a computational machine learning algorithm employing blood and cognitive biomarkers, and that their integration in a multi-domain outperforms algorithms based in only one domain. Independent studies are needed to validate these findings.

## Introduction

Precision psychiatry is attracting increasing attention lately as a recognized priority. One of the goals of precision psychiatry is to develop tools capable of aiding a clinically informed psychiatric diagnosis objectively^1^. A particularly challenging problem faced by clinicians is the lack of objective, quantitative diagnostic tools to establish differential diagnosis of bipolar disorder (BD) or schizophrenia (SZ)^2–5^. Both disorders have symptoms that overlap considerably and share genetic and environmental risk factors^6^, for instance, both can present with psychotic symptoms, alterations in cognition and alterations in cytokines, and acute mania can be very difficult to clinically differentiate from acute SZ. A critique of standard psychiatric diagnosis is the absence of a valid signature of biological and cognitive abnormalities to inform diagnosis^1^. In fact, this is one of the major limits of traditional, phenotypic-based psychiatry which today are based on a descriptive collection of behaviors without objective markers to confirm diagnosis^1^. It is now broadly accepted that cognitive, inflammatory, and immunological factors are altered in both BD and SZ^7^, however, most of these alterations do not respect diagnostic boundaries from a phenomenological perspective and possess great variability in different individuals with the same phenotypic diagnosis and, consequently, none so far has proven to have the ability of reliably aiding in the differential diagnosis of BD and SZ^1,8^. More critically, these biomarkers related to cognition, inflammation and immunology are known to be altered at a group level in both BD and SZ but the extent of these alterations at an individual level is far less characterized. The latter is a pivotal point, since the clinically relevant question is not if patients with BD or SZ in general have these biomarkers altered, but if a particular, individual patient at an individual level, has these alterations.

One important task that has to be carried out for this goal to be achieved is the analysis of how cognitive and immune markers in peripheral blood behave at an individual level across different nosological categories. Given the complexity of psychiatric disorders, it is unlikely that a single biomarker, or even several biomarkers pertaining to the same unit of analysis within a given biological system, i.e., a single domain, will have good enough diagnostic properties to aid real life clinical decisions. However, the analysis of several potential biomarkers assessed simultaneously related to several units of analysis with different levels on information in more than one domain, such as a probabilistic multi-domain data integration approach consisting of cognitive and peripheral blood-based inflammatory and immunological factors, in unison as predictors of differential diagnosis of BD and SZ in an agnostic fashion, possibly helps with this task. From a traditional, inferential statistics paradigm, this is complicated, since inferential statistics cannot appropriately handle high dimensional data. The feasibility of making more accurate predictions using a myriad of complex non-linear high-dimensional interactions that might inform prediction is now facilitated by machine learning methods, which are well-suited for the identification of subtle patterns of information in the data and, as a consequence, are useful to better predict diagnosis at the individual patient level^1^.

In this study, we applied, we believe for the first time, machine learning techniques to two different domains previously described as altered in BD and SZ at a group level—cognitive and peripheral blood-based immune-inflammatory biomarkers—to assess their predictability in ascertaining a differential diagnosis of BD or SZ, in individual patients, with sufficient clinical validity and utility. Our overarching goal is to develop a multi-domain algorithm-based biosignature with good enough diagnostic properties to be able to inform clinical decisions pertaining to the differential diagnosis of BD and SZ in a meaningful way.

## Methods

Inpatients and outpatients between 18 and 65 years, meeting *DSM-IV* criteria (American Psychiatric Association, 1994), for BD or SZ, were consecutively recruited at a university-affiliated psychiatric department (Mondor hospital, University of Paris-Est, Créteil, France) after approval by a French ethic committee and after written informed consent. Controls were included via a clinical investigation center, also in Créteil, France (Center for Biological Resources, Mondor hospital, Créteil, France).

### Inclusion and exclusion criteria

Exclusion criteria for patients and controls were current or past immunosuppressive treatment; recent infection or ongoing inflammatory disease, such as arthritis ankylosing spondylitis, Crohn disease, asthma, or systemic lupus erythematous; a positive serology for HIV-1/HIV-2 or hepatitis A, B, or C; or a comorbid neurologic disorder with cognitive impairment, such as multiple sclerosis, Parkinson disease, head injury, cerebrovascular accident, or Alzheimer’s disease. Healthy controls were included after checking for the absence of personal or first-degree family history of psychiatric disorder and without a personal or family history of autoimmune diseases, inflammatory or infectious past history. Patients were interviewed with a French version of the Diagnostic Interview for Genetic Studies (DIGS) for the assessment of lifetime clinical characteristics of their psychiatric disorder as well as for demographic characteristics. At inclusion, manic symptoms were assessed with the Young Mania Rating Scale (YMRS) and depressive symptoms with the Montgomery-Asberg Depression Rating Scale (MADRS) for BD. Participants with SZ were evaluated using Positive and Negative Syndrome Scale (PANSS). To be included, BD participants had to be in outpatients and in a stable status defined by YMRS score <8 and MADRS score <12, while SZ participants had to have a PANSS score <60.

The cognitive evaluation was conducted in ambulatory care; while for inpatients (also reaching YMRS < 8, MADRS < 12, and PANSS < 60); blood sampling was done very close to the cognitive assessment. Patients were interviewed with a French version of the “Diagnostic Interview for Genetic Studies” (DIGS, 1994) for the assessment of lifetime clinical characteristics of BD and SZ as well as for demographic characteristics (i.e., education level, working status, season of birth, birth place/country). Current medications as well as hospitalization status were recorded.

### Blood-based immunological biomarker profiling

All laboratory analyses were done by personnel blinded to diagnosis status.

#### Serological testing for immunoglobulins (IgGs)

Total IgG, IgA, and IgM were quantified by immunoturbidimetry using commercially available immunoassay reagents (COBAS). IgG sub-classes, i.e., IgG1, IgG2, IgG3, and IgG4 levels were determined on a SPAPLUS analyzer (The Binding Site, Birmingham, UK) using commercially available kits (The Binding Site, Birmingham, UK).

#### Other immune and inflammatory biomarkers

C-reactive protein (CRP) serum level was measured by nephelometry using the cardio-phase high-sensitivity CRP (hs-CRP) kit (Siemens, Germany). Anti-nuclear antibodies (ANA) were detected by indirect fluorescent antibody method on hep2000 cells (Immuno Concepts Inc., CA, USA). Quantification of anti-double strand DNA (anti-dsDNA) antibodies was performed using enzyme-linked immunosorbent assay (ELISA) (anti-ADN-NcX IgG kit; Euroimmun AG, Lübeck, Germany). Anti-extractable nuclear antibodies (anti-centromere CENP-B, anti-JO1, anti-RNP, anti-Scl70, anti-Sm, anti-SSA/Ro, and anti-SSB/La antibodies), anti-phospholipids i.e., anti-cardiolipin (aCL) and anti-β2GP1 (IgG and IgM antibodies as well as anti-Anti-Cyclic Citrullinated Peptide (CCP) were both analyzed using the multiplex immunoassay method (BioPlex™ 2200 Anti-Nuclear Antibody Screen; Bio-Rad Laboratories Inc., France). Anti-neutrophil cytoplasmic antibodies (ANCA) were detected by indirect immunofluorescence (Inova diagnostics, USA) and in case of positivity, specificity (anti-PR3, anti-MPO, anti-elastase, anti-lactoferrin, anti-cathepsin G, and anti-bactericidal/permeability increasing protein [BPI] antibodies) was characterized using the ANCA-Profile ELISA kit (Euroimmun AG, Lübeck, Germany). Rheumatoid factor (RF) was evaluated on the turbidimetric SPAPLUS (Binding Site, Birmingham, UK) Anti-myelin-associated glycoprotein (MAG) IgM and anti-myelin IgM autoantibodies presence were tested by ELISA (BÜHLMANN laboratories AG, Switzerland) and indirect immunofluorescence on slide by (IMMCO diagnostic, NY, USA), respectively.

#### Serological testing for *Toxoplasma gondii* exposure

On enrollment, solid-phase enzyme immunoassay was performed to assess IgG, IgM, and IgA antibodies against *Toxoplasma gondii* as previously described^9^. Qualitative (positive/negative) assay was performed at the Stanley Laboratory of Developmental Neurovirology, Johns Hopkins School of Medicine, Baltimore, Maryland, USA.

### Cognitive data

We employed the Wechsler Adult Intelligence Scale (WAIS) 3rd Edition, which provides a measure of general intellectual function in older adolescents and adults. Seven subtests short forms were used to estimate the full scale IQ (FSIQ), verbal IQ (VIQ), and performance IQ (PIQ), and allowed exploration of the following cognitive areas: picture completion (visual exploration and detail perception), digit-symbol coding (visual-motor coordination, motor, and mental speed), similarities (abstract verbal reasoning), arithmetic (mathematical problem solving), matrix reasoning (nonverbal abstract problem solving, inductive spatial reasoning), digit span (attention, working memory, and mental control), information (general information acquired from culture, semantic memory). An additional subtest, letter-number sequencing, was administered, which along with two other primary subtests, digit span and arithmetic.

The California Verbal Learning Test (CVLT) was also employed. It is designed to measure episodic verbal learning and memory using a multiple-trial list-learning task. The examiner reads the word list and records the patient’s oral responses verbatim in the order in which they are given. It assesses measure rate of learning, learning strategy, short-term and long-term retention and retrieval, recall errors, interference effects and ability to profit from learning cues. Learning efficiency, strategies, interference management and learning bias are measured. A validated French version of the task was used in this study.

Finally, we applied the National Adult Reading Test (NART), which provides an estimate of premorbid intellectual and general cognitive ability level from a word reading test, which provides an estimate of vocabulary size. The participant was required to correctly pronounce the list of NART words presented on a computer screen. In this study, the French version of the NART was used^10^.

### Machine learning strategies and data analysis

#### Data pre-processing

We undertook a complete cases approach, including only participants without missing observations. Usually, when a participant had a variable related to cognition missing, the participant had all variables related to cognition missing, because the cognitive battery was not applied. The same holds true for the blood biomarkers. In this study, 102 blood-based biomarkers and 19 cognitive biomarkers were initially selected for analysis. Among them, any biomarker that was missing in more than 30% of the participants were removed; within each selected biomarkers, subjects with any missing biomarker were also removed. This filtering resulted in a total of 416 participants, being 323, 372, and 279 subjects for blood, cognition and combined biomarkers analysis, respectively, with 27 blood biomarkers and 19 cognitive biomarkers selected for final analysis. Therefore, in the selected set there were no missing values and no imputation process was required. All cognitive and blood biomarkers variables were converted into *z*-scores.

#### Univariate and multivariate analysis

For univariate analysis, nonparametric (Kruskal–Wallis) and chi-square tests were used to identify variables with significant *p*-values for continuous and categorical variables, respectively. In case of the three groups comparison, Tukey–Kramer post-hoc test was performed to check significance between different pairs. For continuous variables, mean ± SD (standard deviation) values were showed to portray the distribution of the variables across different groups. Percentage of positive results (having value 1) was reported for categorical variables.

With the data recorded we built a predictive model, and internally cross-validated the model. Principal component analysis (PCA) and partial least squares discriminant analysis (PLS-DA) were used for multivariate analysis. PCA is used to identify new variables, called principal components, which are a linear combination of the original variables. The new variables are uncorrelated to each other and they are sorted based on their capacity to describe the variance of the original signal^11^. Thus, the first principal component describes the largest variation of the original signal and the last one the smallest. In this study, we applied PCA to extract uncorrelated principal components from the blood-based and cognitive biomarkers to study their capacity to differentiate one group from another in each of the three pairs (BD vs. controls, SZ vs. controls, and BD vs. SZ).

PLS-DA can be regarded as a linear two-class classifier. We used the PLS1 algorithm^12^, where there are always two diagnostic groups (i.e., BD vs. controls, SZ vs. controls, and BD vs. SZ), or pairs of classifiers, of samples and the aim was to measure the importance of variables. The variable influence on projection (VIP) was then calculated, which is the measure of the contribution of each variable according to the variance explained by each PLS component^12^. Once VIP values were calculated, they were used for selecting variables for classification. In this study, we selected the six variables with the top VIP values for classifying BD vs. controls, SZ vs. controls, and BD vs. SZ, which was verified to be the optimum number of variables in our models that maximized the discriminant properties without unnecessarily overcomplicating, and therefore overfitting, the models. Six is also the number selected as the optimum number to balance practical usability with model performance.

#### Validation and area under the curve (AUC) calculations

We built linear discriminant (LD) binary classifiers for three diagnosis pairs: BD vs. controls, SZ vs. controls, and BD vs. SZ. We used the 10-fold cross-validation strategy. In this strategy, the data is randomly divided into ten equal parts. For each fold, one part is used as validation set and the rest as training set. The process was repeated for all ten parts so that the union of validation sets equals the original data set. The prediction on the ten validation sets is then combined. We reported the AUC value as the predictive measure of the final model. Receiver operating characteristic (ROC) plots^13^ were derived from linear discriminative analysis (LDA) based on the top six biomarkers from the PLS-DA approach. Three different models according different domains were developed using selected biomarkers from blood (single immune-inflammatory factors in the peripheral blood domain), cognition (single cognitive domain) and their combined pool (multi-domain composed of immune-inflammatory factors in peripheral blood and cognitive biomarkers). The summary index of the optimal sensitivity and specificity—when both sensitivity and specificity are maximized (summary index equals positive predictive value plus negative predictive value minus 100)—of the set of selected biomarkers for the three models were determined by the ROC curve and the AUC^13^. In a general situation, an AUC of 0.90–1.0 is regarded as very high (excellent), of 0.80–0.89 high (good), of 0.70–0.79 moderate (fair), of 0.60–0.69 low (poor), and of 0.50–0.59 as very low (fail or useless)^14^. In addition, based on the ROC curves, we determined the true positive rate (TPR) (sensitivity), the true negative rate (TNR) (specificity), the false positive rate (FPR) (1—specificity), and the false negative rate (FNR) (1—sensitivity)^15^. To better analyze clinical validity and utility, we calculated the positive predictive value (PNV) and the negative predictive value (NPV) in different simulated settings with different prevalence^16,17^. All analyses were performed in Matlab R2014b.

We conducted this study according to the STARD statement (Standards for reporting diagnostic accuracy)^18,19^ and transparent reporting of a multivariable prediction model for individual prognosis or diagnosis (TRIPOD)^20,21^ statement.

### Ethical aspects

The study was carried out in accordance with ethical principles for medical research involving humans (WMA, Declaration of Helsinki). The assessment protocol was approved by the relevant ethical review board. All data were collected anonymously. As this study include data coming from regular care assessments, a non-opposition form was signed by all participants.

## Results

The total population sample consisted of 416 participants. Twenty-seven blood-based biomarkers and 19 cognitive biomarkers were selected for final analysis after biomarkers with missing data, as defined in the “Methods” section, were excluded. This resulted in 323 persons contributing to the blood-based domain (121 with BD, 71 with SZ, and 131 controls), 372 persons contributing to the cognitive domain (117 with BD, 84 with SZ, and 171 controls), and 279 persons contributing to the multi-domain composed by the immune blood-based domain plus the cognitive domain (98 with BD, 58 with SZ, and 123 controls). Characteristics of the sample are shown in Table 1.

**Table 1:** Demographic characteristics of the sample with both immune blood-based and cognitive biomarkers.

Characteristic	Bipolar disorder *N* = 98	Schizophrenia *N* = 58	Control *N* = 123	*p*
Masculine sex^1^	50 (51.02)	43 (74.14)	49 (39.84)	**<0.001**
Age^2,a,b,c^	45.14 ± 12.93	35.26 ± 10.97	39.19 ± 13.17	**<0.001**
Age of onset of the disorder^2^	26.86 ± 10.53	24.05 ± 7.11	NA	0.074
Length of illness^2,a^	18.13 ± 12.26	11.21 ± 9.36	NA	<**0.001**
BMI^2^	24.69 ± 3.76	25.12 ± 5.56	24.43 ± 4.16	0.605
Smokers^1^	20 (20.41)	5 (8.62)	18 (14.63)	0.124
YMRS^2,a^	3.96 ± 1.65	4.66 ± 3.38	NA	**0.003**
MADRS^2^	6.19 ± 4.88	7.89 ± 6.20	NA	0.151
PANSS total^2,a^	37.38 ± 13.21	55.69 ± 14.65	NA	<**0.001**
PANSS positive total^2,a^	7.98 ± 2.85	12.52 ± 6.62	NA	<**0.001**
PANSS negative total^2,a^	9.08 ± 4.50	18.87 ± 9.03	NA	<**0.001**
History of suicide attempt^1,a^	39 (39.80)	19 (32.76)	NA	<**0.001**
Current hospitalization^1,a^	25 (25.51)	26 (44.83)	NA	<**0.001**
Drug-free^#,1,a^	4 (4.08)	2 (3.45)	NA	<**0.001**

^1^Chi-square test. Values shown as raw numbers and (%).

^2^Values shown as mean and standard deviation. Bipolar disorder, schizophrenia, and controls, analyses by analysis of variance (ANOVA) followed by Tukey-Kramer post-test. Bipolar disorder and schizophrenia, analyses by *t*-test.

^a^Bipolar disorder is significantly different from Schizophrenia.

^b^Bipolar disorder is significantly different from control.

^c^Schizophrenia is significantly different from control.

^#^Drug-free defined as without use of psychiatric medication for at least 7 days.

*NA* not applicable.

### Univariate analysis

Univariate analysis was carried out to identify single blood biomarkers and cognitive biomarkers signals that were associated with disease status and to establish information and methodology applicable to multivariate approach in subsequent analysis.

Results from nonparametric tests showed significant differences between the BD and control pair, between the SZ and control pair of classifiers, and also between the BD and SZ pair, with larger differences observed for participants with SZ related to the cognitive biomarkers and for BD with the immune blood biomarkers (Supplementary Table 1). Supplementary Fig. 1 show the relative difference (fold changes with confidence intervals) for each single biomarker when comparing participants with BD versus controls and participants with SZ versus controls. To obtain a common scale, all results presented are based upon log transformed data.

### Multivariate analysis

Multivariate analysis was also carried out to explore correlations within the dataset, and to identify whether multiple analytes could increase the discrimination between cases and controls. The analysis was performed in two stages: (1) principal components analysis (PCA) for unsupervised analysis of the full dataset, aimed at determining whether a multivariate signal was present; (2) partial least squares discriminant analysis (PLS-DA) to help determine the identity of the biomarkers responsible for the separation.

Supplementary Fig. 2 shows the PCA plots obtained. Similar to the results of the univariate analysis, separation can be observed, in particular for SZ and BD from controls, for both blood-based biomarkers and for cognitive biomarkers. It should be noted that these graphs were produced by PCA without previous disease classification information, in contrast to the partial least squares (PLS) approach, which is known to split classified groups even from random data sets. Having established a separation by PCA, the step of PLS-discriminant analysis was used solely to compute a series of scores (VIPs) to assess the contribution of individual blood-based and cognitive biomarker to these dimensions.

The contribution of each blood or cognitive biomarker to the separation of participants with BD or SZ from controls, according the VIPs found, were computed for SZ and BD, to derive the most informative biomarkers. For the immune blood-based biomarkers, IgG1, IgG2, IgG3, and anti-cardiolipin antibodies A (ACA A) showed the best properties for discriminating BD from controls, and cytomegalovirus (CMV), herpes simples virus 2 (HSV2), and *Toxoplasma Gondii* for SZ from controls. For the cognitive biomarkers, WAIS deterioration showed the best discriminant properties for BD from controls, and CVLT total number of correct answers during list a short delay cued recall (CVLT SDCR) and CVLT total number of correct answers during list a long delay cued recall (CVLT LDCR) for SZ from controls. WAIS digit symbol coding (WAIS DSC) also showed significant discriminant capacity, however less than the others described above. When considering the capacity of both domains together in separating BD from controls and SZ from controls, the peripheral blood-based biomarkers domain showed better discriminating properties for BD and the cognitive domain showed better discrimination for SZ.

Therefore, the blood-based biomarkers that were needed for the multi-domain model are IgG1, *Toxoplasma gondii* IgG, and anti-cardiolipin antibodies (ACA). The cognitive biomarkers are related to all batteries applied (WAIS, CVLT, and NART33). The biomarkers that contributed to the algorithms of all three final models are on Table 2.

**Table 2:** Biomarkers contributing to the multi-domain model composed of immune blood-based and cognitive biomarkers.

Model	BD vs. CT	SZ vs. CT	BD vs. SZ
Single-domain	IgG1	HSV2	IgG1
Immune blood biomarkers	IgG3	*Toxoplasma gondii* IgM	HSV2
	Anti-cardiolipin	CMV	IgG4
	IgG2	IgG2	*Toxoplasma gondii* IgM
	Anti-TTGG	IgG	Anti-gliadin A
	Anti-CCP	ANCA	Anti-TTGG
Single-domain	WAIS deterioration	WAIS DSC	WAIS DSC
Cognitive biomarkers	WAIS DSC	CVLT LDCR	CVLT LDCR
	WAIS inform	CVLT SDCR	WAIS DS
	NART33 IQ	WAIS deterioration	CVLT SDCR
	WAIS Let-Num Seq	WAIS Let-Num Seq	NART33 IQ
	CVLT LDCR	WAIS DS	WAIS BD
Multi-domain	WAIS deterioration	WAIS DSC	WAIS DSC
Immune blood and	WAIS DSC	CVLT LDCR	IgG1
cognitive biomarkers	IgG1	CVLT SDCR	WAIS DS
	*Toxoplasma gondii* IgG	WAIS deterioration	WAIS Obj Ass
	Anti-cardiolipin	WAIS arithmetic	CVLT LDCR
	WAIS inform	WAIS Let-Num Seq	NART33 IQ

*WAIS DSC* WAIS digit symbol coding total score, *WAIS Inform* WAIS information total score, *CVLT SDCR* CVLT total number of correct answers during list a short delay cued recall, *CVLT LDCR* CVLT total number of correct answers during list a long delay cued recall, *WAIS DS* WAIS digit spam total score, *WAIS Obj Ass* WAIS object assembly total score, *NART33 IQ* NART 33 items total IQ score, *WAIS BD* WAIS block design total score.

We wanted to verify if a multi-domain approach considering both the immune blood biomarkers and the cognitive domains would produce an algorithm with better diagnostic properties than each of the domains in isolation. To assess the capability of our diagnostic set of markers to discriminate correctly between cases and controls, we derived ROC plots based on a linear discrimination analysis model (LDA) built upon the six markers with the highest contribution as determined by PLS-DA.

### Predictive diagnostic properties and clinical validity and utility of the algorithms for the immune blood-based, cognitive, and combined domains

#### General predictive diagnostic properties

The general diagnostic predictive properties of each domain varied in the three diagnostic pairs of classifiers. The general accuracy was slightly lower than the AUC for all three models and all pairs of classifiers.

The single immune blood-based domain showed a moderate AUC and sensitivity for discriminating BD from controls, SZ from controls, and BD from SZ. The specificity, however, was low in all three pairs.

The single cognitive domain showed better predictive properties for separating BD from controls than the single immune blood-based domain, with a high AUC and moderate sensitivity and specificity. The predictive properties of the cognitive domain was also better than in the immune blood-based domain for separating SZ from controls, with all three parameters showing high discrimination of SZ from controls. Regarding the SZ vs. BD pair, the parameters were all of moderate value.

**Fig. 1:**
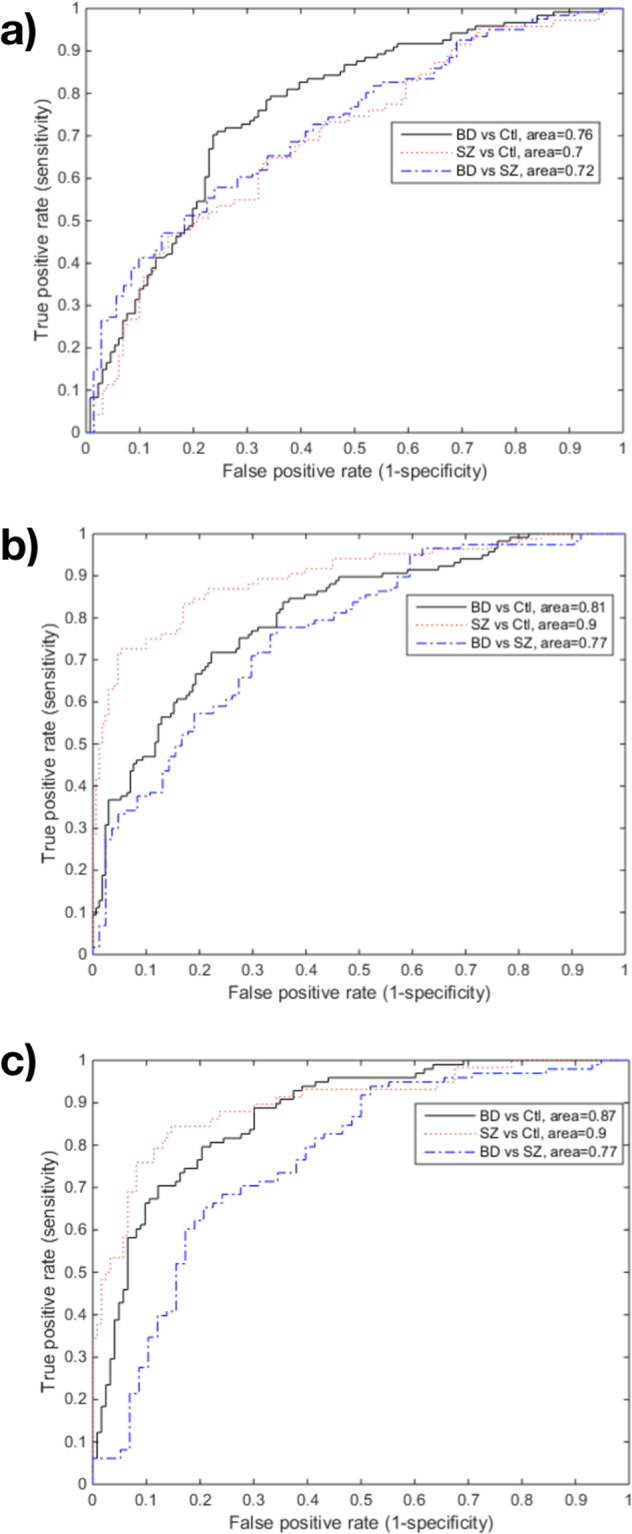
Receiver operating characteristics (ROC) plot derived from the linear discriminate analysis (LDA) using blood-based markers based on the top finding from PLS approach. **a** Receiver operating characteristics (ROC) plot derived from the linear discriminate analysis (LDA) using blood-based markers based on the top finding from PLS approach. ROC plot of sensitivity (true positive rate) vs. 1-specificity (false positive rate) based on a LDA build upon the six markers with the highest contribution as determined by the PLS discriminant analysis. **b** ROC plot derived from the LDA using cognitive markers based on the top finding from PLS approach. ROC plot of sensitivity (true positive rate) versus 1-specificity (false positive rate) based on a LDA build upon the six markers with the highest contribution as determined by the PLS discriminant analysis. **c** ROC plot derived from the LDA using blood-based markers and cognitive markers based on the top finding from PLS approach. ROC plot of sensitivity (true positive rate) vs. 1-specificity (false positive rate) based on a LDA build upon the six markers with the highest contribution as determined by the PLS discriminant analysis.

Finally, the model combining both the immune blood-based and the cognitive domains was superior in correctly predicting BD from controls than the models considering each domain in isolation, with a high AUC and sensitivity and a moderate specificity. However, its predictive properties were exactly the same as the single cognitive domain in separating the SZ from control groups, and only slightly better for discriminating the BD from SZ groups than the cognitive domain (Fig. 1a–c and Table 3).

**Table 3:** Predictive value of the final models: area under the curve and discrimination parameters of the models obtained by the three linear discriminant (LD) binary classifiers.

	**BD vs. CT**	**SZ vs. CT**	**BD vs. SZ**
*Single domain—immune blood*			
General AUC	0.73	0.71	0.75
Accuracy	69.17%	70.30%	71.73%
True positive rate (Sensitivity)	72.31%	71.48%	79.10%
True negative rate (Specificity)	64.39%	61.27%	64.49%
False positive rate (1—Specificity)	35.61%	38.73%	35.51%
False negative rate (1—Sensitivity)	27.69%	28.52%	20.90%
*Single domain—cognition*			
General AUC	0.81	0.90	0.77
Accuracy	75.35%	87.06%	72.14%
True positive rate (Sensitivity)	76.12%	84.43%	71.39%
True negative rate (Specificity)	71.19%	81.14%	71.27%
False positive rate (1—Specificity)	28.81%	18.86%	28.73%
False negative rate (1—Sensitivity)	23.88%	15.53%	28.61%
*Multi-domain—immune blood* *+* *cognition*			
General AUC	0.86	0.89	0.80
Accuracy	79.73%	86.18%	76.43%
True positive rate (Sensitivity)	88.29%	84.46%	71.29%
True negative rate (Specificity)	71.11%	81.39%	73.33%
False positive rate (1—Specificity)	28.89%	18.61%	26.67%
False negative rate (1—Sensitivity)	11.71.%	15.54%	28.71%

AUC sensitivity (true positive rate), specificity (true negative rate), and false positive (1—specificity) and false negative (1—sensitivity) rates. Receiver operating characteristic (ROC) plots were derived from linear discriminative analysis (LDA) based on the top six biomarkers from the PLS-DA approach. Three different pairs of classifiers (BD vs. control, SZ vs. control, and BD vs. SZ) were analyzed using selected biomarkers from blood, cognition, and their combined pool. Optimal sensitivity and specificity of the set of selected biomarkers for the three models and were determined by the ROC curve and the AUC. An AUC of 0.90–1.0 is very high (excellent), of 0.80–0.89 high (good), of 0.70–0.79 moderate (fair), of 0.60–0.69 low (poor), and of 0.5–0.59 is very low (fail or useless). Color code: “success” showed in green, moderate “success” showed in yellow, “failure” showed in red.

*AUC* area under the curve, *BD* bipolar disorder, *SZ* schizophrenia, *CT* control.

#### Clinical usefulness and validity of the multi-domain and cognitive-domain predictions algorithms as diagnostic tests

**Fig. 2:**
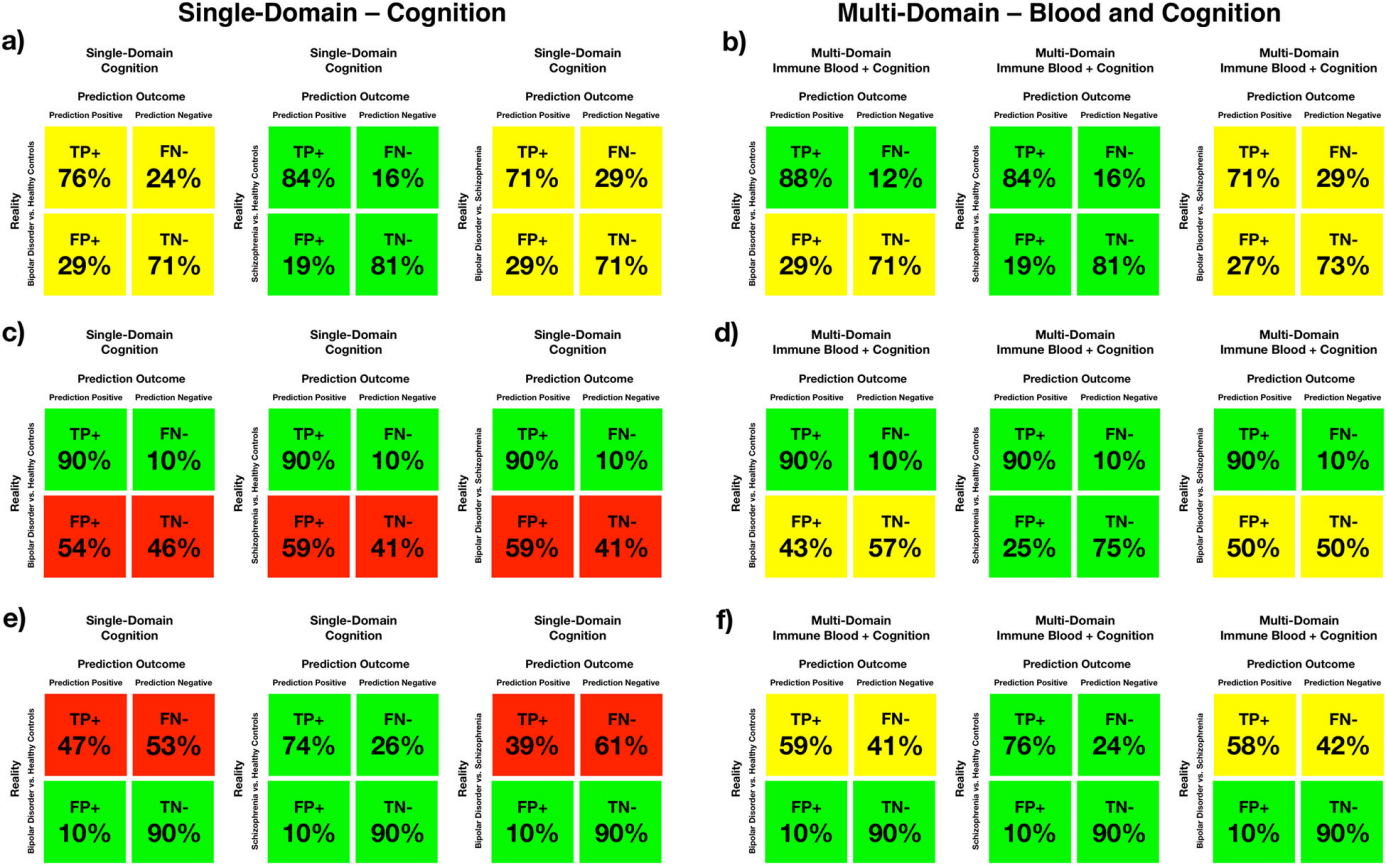
True positive rate (TPR), true negative rate (TNR), false positive rate (FPR) and false negative rate (FNR) showed as percentage (%) of the values for the multi-domain of immune blood and cognitive biomarkers predictions for the bipolar disorder vs. control pair, for the Schizophrenia vs. control pair, and for the bipolar disorder vs. Schizophrenia pair. **a**, **b** General performance maximizing both sensitivity and specificity. The predictions were categorized as “success” when the predictions of TPR and FNR were based on high sensitivity (i.e., 80% or more) and also as “success” when the predictions of TNR and FPR were based on high specificity (i.e., 80% or more). The predictions were categorized as “moderate success” when the predictions of the above were based on moderate sensitivity or specificity (i.e., from 70 to 79%), and the predictions were categorized as “fail” when these predictions were based in low or very low sensitivity or specificity (i.e., 69% or less). **c**, **d** High sensitivity (90%) scenario. The predictions were categorized as “success” when the FPR was below 30%, as a “moderate success” when those values were from 31 to 50%, and as “failure” when those values were higher than 50%. **e**, **f** High specificity (90%) scenario. The predictions were categorized as “success” when the FNR was below 30%, as “moderate success” when those values were from 31 to 50%, and as “failure” when those values were higher than 50%. Color code: “success” showed in green, “moderate success” showed in yellow, and “failure” showed in red.

In clinical practice, a clinical test with high diagnostic properties is considered useful, and therefore worth ordering for a patient, while a test with moderate diagnostic properties is possible useful and a clinician would consider ordering it depending on the circumstances, and a test with low or very low diagnostic properties is considered without clinical value, and, consequently, should not be ordered in the great majority of cases^15,22^. Taking this into consideration, we classified the algorithms predictions based on the multi-domain (the one with the best properties) and in the cognitive domain (the second best) as “success”, “moderate success”, or “failure”, [i.e., with high (≥80%), moderate (70–79%), or low (<70%) diagnostic properties, respectively]^14^. The general predictions of both models were a “success” as a possible test, in a scenario in which both sensitivity and specificity are maximized, for the SZ vs. control pair, and a “moderate success” for the BD vs. SZ pair. However, for the BD vs. control pair, the multi-domain model was a “success” regarding sensitivity (the TPR), while both models were a “moderate success” regarding specificity (the TNR) (Fig. 2a, b).

#### Predictive diagnostic properties and clinical validity in very high sensitivity or very high specificity scenarios

Depending on the clinical situation, a clinician might consider a diagnostic test more useful if it has a high sensitivity (i.e., high TPR) or if it has a high specificity (i.e., high TNR)^15,22^. In order to verify this, we built another two different scenarios, one in which the models would perform with high sensitivity, in which we set the sensitivity at 90%, and one with high specificity, in which we set the specificity at 90%. In both scenarios, we then verified which would be the correspondent specificity in the 90% sensitivity scenario and which would be the sensitivity in the 90% specificity scenario. In these scenarios, it is expected that the counterpart sensitivity or specificity will be lower, and, therefore, the test can be considered a “success” when the correspondent sensitivity or specificity is at least 70%, as a “moderate success” when it is from 50 to 69%, and a “failure” when it is below 50%^15,22^. In both scenarios, the algorithm of the multi-domain model composed of immune blood and cognitive biomarkers outperformed the algorithm of the single-domain composed only for the cognition model (Fig. 2c–f).

#### Clinical utility in scenarios with different prevalence

The most clinically relevant diagnostic information that a diagnostic test can provide is the PPV and the NPV, which better inform clinical utility. These parameters are highly dependent on the prevalence of the condition of interest in a given clinical setting^17^. In order to calculate the PPV and the NPV, we simulated nine different clinical settings with prevalence ranging from 10 to 90%. Thereafter, we calculated these values considering the multi-domain algorithms for all three pairs of classifiers in the three different scenarios (i.e., balanced sensitivity and specificity, high sensitivity, and high specificity). In a clinical setting with a relatively low prevalence ranging from 10 to 30%, the NPV varied from 83 to 98%, and the PPV from 17 to 77%; the scenarios with balanced prevalence and with high specificity performed better, with the high sensitivity scenario having no advantage over the other two; the scenario with high specificity showed the highest rates of PPV. In a clinical setting with a relatively high prevalence ranging from 70 to 90%, the PPV varied from 86 to 99%, and the NPV from 18 to 76%; the scenarios with balanced prevalence and with high sensitivity performed better, with the high specificity scenario having no advantage over the other two; the scenario with high sensitivity showed the highest rates of NPV. In clinical settings with a moderate prevalence of 40–60%, all three scenarios are useful, with different advantages. As expected, the high sensitivity scenario showed the highest NPV, and, conversely, the high specificity scenario showed the highest PPV (Table 4).

**Table 4:** Positive and negative predictive values of the multi-domain model in different simulated settings with different prevalence.

Pair of classifier in	Prevalence (%)								
Different scenarios	10	20	30	40	50	60	70	80	90
*Balanced Se and Sp*									
BD vs. SZ—*PPV*	23	40	53	64	72	80	86	91	96
BD vs. SZ—*NPV*	96	91	85	65	72	63	52	39	22
BD vs. HC—*PPV*	25	43	57	67	75	82	88	92	93
BD vs. HC—*NPV*	98	96	93	90	86	80	72	60	60
SZ vs. HC—*PPV*	32	53	65	75	82	87	91	95	93
SZ vs. HC—*NPV*	96	95	92	88	84	77	61	56	36
*High Se—90%*	10	20	30	40	50	60	70	80	90
BD vs. SZ—*PPV*	17	34	44	54	64	73	81	88	94
BD vs. SZ—*NPV*	98	92	92	88	84	77	68	56	36
BD vs. HC—*PPV*	19	34	47	58	68	76	83	89	95
BD vs. HC—*NPV*	98	96	93	90	85	79	71	59	39
SZ vs. HC—*PPV*	29	47	61	71	79	84	89	94	97
SZ vs. HC—*NPV*	99	98	95	92	88	83	76	65	45
*High Sp—90%*	10	20	30	40	50	60	70	80	90
BD vs. SZ—*PPV*	39	59	71	79	85	90	93	94	98
BD vs. SZ—*NPV*	95	90	83	76	68	59	48	35	18
BD vs. HC—*PPV*	40	60	72	80	86	90	93	96	98
BD vs. HC—*NPV*	95	90	84	77	69	59	48	35	20
SZ vs. HC—*PPV*	46	66	77	84	88	92	95	97	99
SZ vs. HC—*NPV*	97	94	90	85	79	71	62	48	29

## Discussion

We developed a probabilistic multi-domain data integration model consisting of immune and inflammatory biomarkers in peripheral blood and cognitive biomarkers using machine learning to predict diagnosis of BD and SZ, two severe psychiatric disorders that have a differential diagnosis that can be challenging^23^. This is particularly true for the diagnosis of BD and for the differential diagnosis of BD and SZ^2–5,14^. Our multi-domain model performances for the BD vs. control (sensitivity 80% and specificity 71%) and for the SZ vs. control (sensitivity 84% and specificity 81%) pairs are high in general, and are relatively similar to other diagnostic tests already employed in general clinical practice. Just in order to make a comparison, in multiple sclerosis, which, similar to what happens in psychiatry, is a complex condition with heterogeneous presentation and a variety of pathophysiology mechanisms, and that thus might be better called a syndrome rather than a distinct disease, the combination of brain magnetic resonance scan imaging and cerebrospinal fluid analysis have a sensitivity of 84% and a specificity of 74%^24^. However, our multi-domain model has only moderate performance for the differential diagnosis of BD and SZ (sensitivity 71% and specificity 73%). This is the most challenging and crucial differentiation and these findings were somehow expected, since people with BD and SZ share many more similarities than people with one of these disorders share with healthy people;^4,14^ as a matter of fact, other studies that employed peripheral blood biomarkers, neuroimaging, or cognition as single-domains have systematically found this^3,5,14,25–29^.

### Clinical validity and utility

For a machine learning algorithm to be clinical valuable, to show that a test has higher than chance statistical significance is not enough, the prediction has to be high enough to change decision-making in real life^2,3,30,31^. For this reason, we categorized our predictions according to their perceived clinical validity and utility.

In diagnostic medicine, a highly sensitive test is less likely to overlook a positive and a highly specific test is less likely to register a positive in the absence of the disease. Preferably, a test would have both high sensitivity and specificity, with high percentage of true positives and true negatives and, consequently, low percentage of false positives and negatives^16^. Tests with high diagnostic properties are preferable, and usually ordered for a patient, while those with moderate properties are ordered depending on the circumstances. Taking this into consideration, we classified the predictions based on the multi-domain and in the cognitive domain as “success”, “moderate success”, or “failure” (i.e., at least high, moderate, and low diagnostic properties, respectively). In this classification, the multi-domain based predictions were generally superior, albeit not by a large margin, to the single-domain ones, particularly for BD.

In a clinical situation where not missing a positive case is more important, when being inclusive is better (for instance, when deciding if a patient should be kept in a high risk program for further follow-up and observation), it is preferable to maximize sensitivity to the detriment of specificity, and the cutoff of the diagnostic instrument can be set at a value in which the test will have a higher sensitivity. Conversely, in a clinical situation where avoiding to misclassify a person without the condition as having the condition is more important (for instance, mislabeling a patient as having SZ, since diagnosing SZ in the absence of the disease can have deleterious repercussions to the patient) it might be preferable to maximize specificity to the detriment of the sensitivity, and the cutoff of the diagnostic instrument can be set at a value in which the test will have a higher specificity^19^. In the former scenario, our predictions were also a “success” in preserving at least moderate or high specificity, meaning that the test is also a “success” in keeping the misclassification of persons without the condition of interest as persons with the condition relatively low (specificity moderate or high, with low FPR) only in the multi-domain model. In the later scenario, our predictions were also a “success” in preserving at least moderate or high sensitivity, meaning that the test is also a “success” in keeping the misclassification of persons with the condition of interest as persons without the condition relatively low (specificity moderate or high, with low FNR) in both models only for the SZ vs. control pair, and a “success” for the BD vs. control and for the BD vs. SZ pairs only in the multi-domain model. These results mean that the multi-domain models are in general better and more versatile.

Another point to consider when implementing a diagnostic device is the feasibility and practicality of the test. Selecting the top six features, as we did here, as opposed to the full set, increases the clinical applicability, and consequent implementation, of the approach. This means that our algorithms have increased clinical utility. A second advantage is that the laboratory tests used in the final algorithms are available in routine clinical laboratories at the moment, meaning that the long process of developing a test with sufficient laboratory validity can be bypassed. The algorithms related to the multi-domain require the laboratory assessment of only three tests and the application of the WAIS, of the CVLT, and of the NART 33. The employment of laboratory exams and the application of these cognitive batteries might be time consuming, however, considering that BD and SZ are serious and permanent conditions that have life-time consequences, a battery of test capable of predicting a correct diagnosis, even if time-demanding and possible expensive, will likely be cost-effective in the long-term.

### Limitations

Some limitations in our study should be noticed. (1) This is a case-control study; in diagnostic studies with this design, patients with borderline or mild expressions of the disease, and conditions mimicking the disease, are excluded, which can lead to an overestimation of both sensitivity and specificity in what is called spectrum bias because usually only persons with a more severe and well-defined clinical presentation are included, and, therefore, the spectrum of the participants in the study will not be fully representative of the persons seen in most clinical settings where the test will be applied^29,30^. Here, both the SZ and the BD groups had a length of illness of more than 10 years. This signals that people in both the BD and SZ groups probably had a more severe presentation. In addition, there is evidence that both cognition and blood biomarkers can change with the course of the disease, with more accentuated alterations with an increased length of illness. The fact that the mean length of illness in our sample is more than 10 years might have been a confounder, however, we adjusted our analysis for this variable. (2) The selection of the threshold value for the test that maximizes both the sensitivity and specificity of the test may lead to overoptimistic measures of test performance. The performance of this cutoff in an independent set of patients may not be the same as in the original study^32^. (3) The reference test, in this case clinical diagnosis according to DSM-IV, can yield different results over time and in different settings. Field tests of the DSM-IV show pooled test-retest reliability Kappa’s of 0.46 in SZ and 0.56 in BD. In this situation, when there is no perfect gold standard, the diagnostic accuracy of the new test is likely to be biased^15^, however, this is unavoidable in the current psychiatric reality. (4) Since this is a cross-sectional study, we cannot infer causality. There is evidence that immuno-inflammatory markers and cognition are altered since the beginning of these disorders, and it is broadly accepted that inflammation has a causal role in both BD and SZ. However, from a diagnostic perspective, this is irrelevant. A given marker has only to discriminate between two conditions, regardless if it is a cause, consequence, or correlate of the pathophysiological process. Interestingly, the blood biomarkers found altered in BD were more related to inflammation, and the ones altered in SZ were more related to immunology. This is in tandem with Mendelian Randomization reports in which an increase in CRP levels is causally related to BD and that a decrease in CRP levels is causally related to SZ^33^. (5) There is a difference in some of the demographic parameters among the three groups regarding sex, age, length of illness, current hospitalization, and history of suicide attempt, though the results remained unchanged when taking these into consideration as covariates. (6) Ideally, an algorithm created by machine learning techniques for diagnosis should be validated in an independent dataset (the testing dataset). We employed only a training dataset, however, we cross-validated our model internally.

### Future directions for the development of diagnostic tests in psychiatry

There are some considerations that might help to move the Precision Psychiatry field regarding diagnosis in psychiatry and that ideally should be implemented whenever logistically possible. (1) An important consideration in the context of development of medical tests for disease classification is the differentiation between diagnoses. Rather than distinguishing patients with BD or SZ from healthy controls, a substantial part of clinical practice involves laborious and error-prone differential diagnostic processes to distinguish different patient groups from each other rather than from healthy controls. To date, few studies have investigated the potential of biomarkers, either blood-based, cognitive, or neuroimaging, in diagnostic models to differentiate among diagnostic groups, particularly for the crucial differentiation of BD in mania from SZ and unipolar from bipolar depression^1,3,34^. (2) Diagnostic studies should always follow and report their results according to the STARD^18^ and TRIPOD^21^ guidelines to improve quality and transparency of the reports. These guidelines are the diagnostic and prognostic guidelines equivalents of the CONSORT guidelines employed in clinical trials. (3) In most situations, the analysis and computational algorithm created should be done employing machine learning techniques, since usually they outperform traditional inferential statistics. (4) The inclusion of clinical variables into a diagnostic algorithm might improve their discriminatory performance. Also, including patients with a broader variety of clinical presentations, including participantes with a recent onset of the disorder, is desirable. (5) In diagnostic studies in psychiatry, the gold standard against to which any new possible test is compared is a clinical psychiatric diagnosis following DSM or ICD criteria. However, clinical phenotype diagnosis of psychiatric disorders lacks validity and robust test–retest reliability, by the simple reason that the DSM and ICD do not have a valid neurobiological basis. Thus, there is a circularity problem, since a new potential test is compared to an imperfect gold standard. One possible solution to this problem would be to perform an agnostic machine learning technique, such as clustering, to identify biologically informed subtypes of psychiatric disorders, and then develop and test the new diagnostic algorithm against these clusters. (6) Most diagnostic studies published so far report a PPV and a NPV calculated according to the number of cases and number of controls included. Yet, this is usually inappropriate, particularly in studies employing convenience samples, because the prevalence of cases in the study is artificial and almost never reflects the real, clinical prevalence. The PPV and the NPV are highly influenced by the prevalence, and its calculations based on an artificial prevalence will not be informative and are almost always misleading. This is a difficult problem, however, because the prevalence of a condition varies in different clinical settings. To account for this, in this study, we chose not to calculate these values based on the artificial prevalence of patients in our sample, but to calculate the PPV and the NPV in several hypothetical different clinical settings with varying prevalence. We consider this more appropriate and suggest future studies to do the same. (7) When developing new diagnostic algorithms, it would be desirable to include in the training dataset as many relevant markers as possible, including neuroimaging. As such, searching for possible new blood biomarkers employing “omics” techniques, such as proteomics and metabolomics, which can facilitate the discovery of several biomarkers simultaneously in a completely agnostic fashion, is desirable^1^.

## Conclusions

In conclusion, our results show, we believe for the first time, that the diagnosis of BD and of SZ, and that the differential diagnosis of BD and SZ, can be predicted with possible clinical utility by a computational machine learning algorithm employing blood and cognitive biomarkers, and that their integration in a multi-domain considering different units of analysis generally outperforms algorithms based in only one domain. Independent studies are needed to validate such findings, particularly studies with longitudinal and consecutively collected samples, and test whether these predictive models might be further enriched by additional clinical, neurobiological, and neuroimaging information.

## Supplementary information

Supplemental Material
