# Supplementary material for: Precision psychiatry with immunological and cognitive biomarkers: a multi-domain prediction for the diagnosis of bipolar disorder or schizophrenia using machine learning
Source: Transl Psychiatry. 2020 May 24;10:162. doi: 10.1038/s41398-020-0836-4 (PMC7246255; doi:10.1038/s41398-020-0836-4)
Supplement: Supplementary file 1 — Supplemental Material [file 41398_2020_836_MOESM1_ESM.pdf]

Diagnosis of Bipolar Disorder and Schizophrenia with blood-based biomarkers and neurocognition: A multi-domain approach using machine learning

Brisa S. Fernandes [1 #,*], Chandan Karmakar [2,3 #], Ryad Tamouza [4,5], Truyen Tran [3], John Yearwood [2], Nora Hamdani [4], Hakim Laouamri [5], Jean-Romain Richard [4,5], Robert Yolken [6], Michael Berk [1,7,8], Svetha Venkatesh [3], Marion Leboyer [4,5 *]

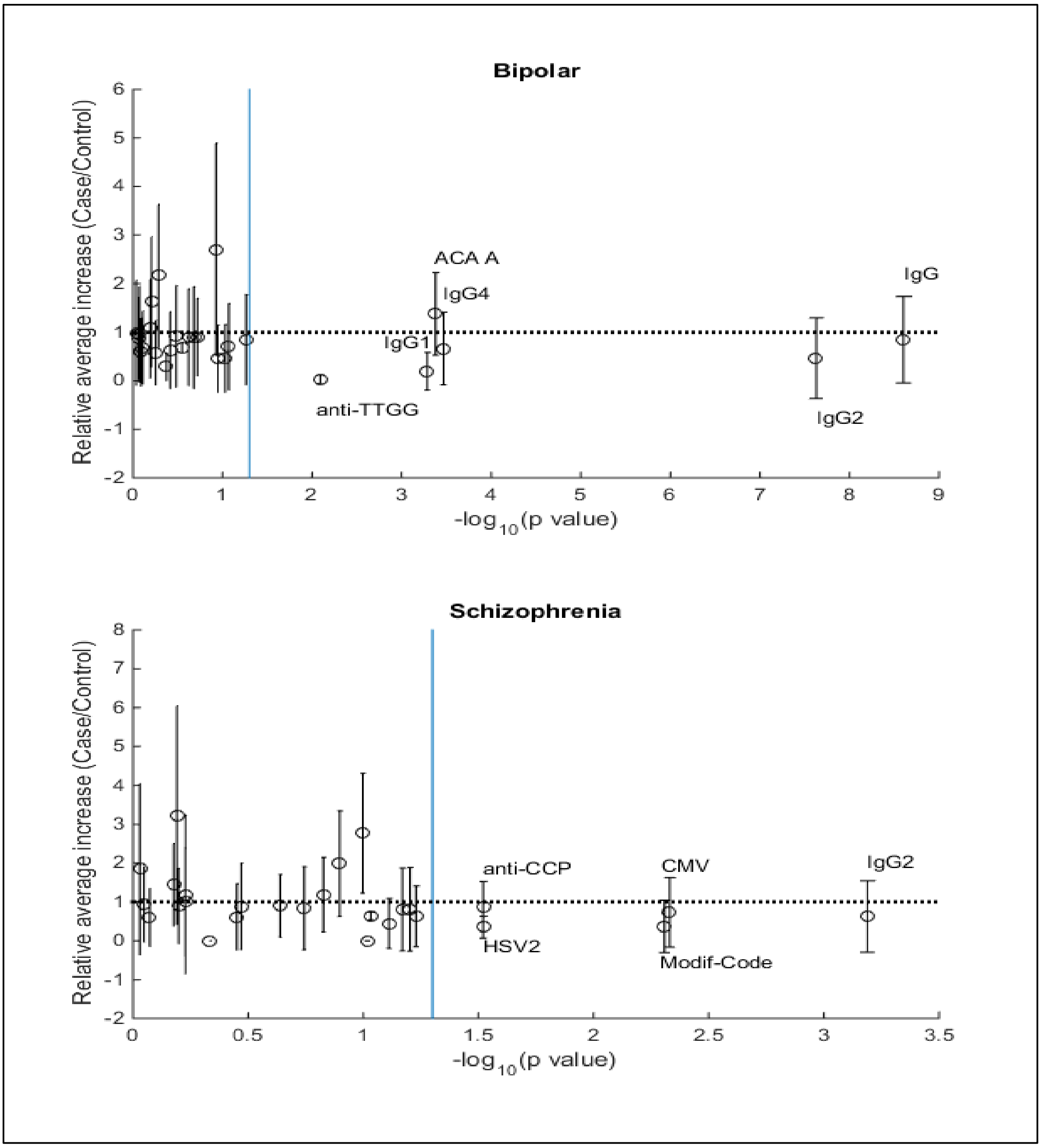

Figure S1. Relative change of blood-based markers in bipolar disorder or schizophrenia against their p-values. Plot of relative changes for measured analytes in bipolar disorder and schizophrenia. The Y axis reports the relative increase (or decrease) as the ratio (and the confidence against their p-values interval) based on analysis of log-transformed data from cases/controls. Reference vertical line corresponds to p-value threshold at a 5% significance level.

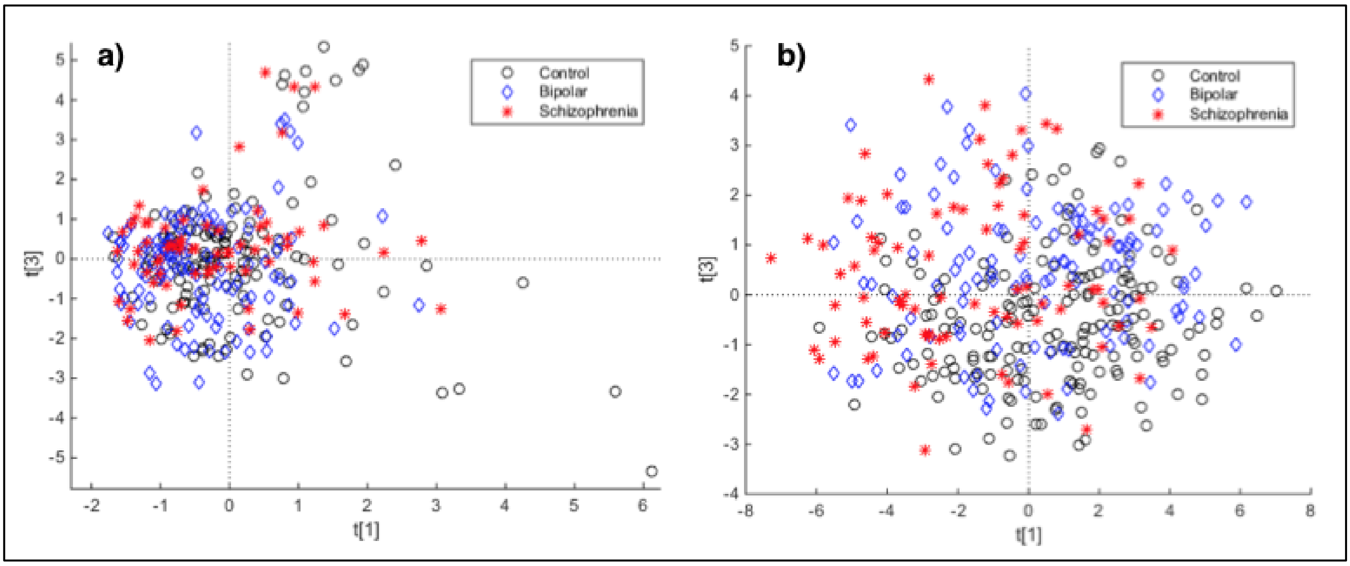

Figure S2. PCA plot showing distribution of samples from control, schizophrenia and bipolar disorder. **a)** PCA plot of blood-based markers, where 1st and 3rd components of the model are shown. Components are chosen to show better discriminatory performance. **b)** PCA plot of cognitive markers, where 1st and 3rd components of the model are shown. Components are chosen to show better discriminatory performance. t [1], first component; t [3], third component.

Table S1. Peripheral blood-based and cognitive biomarkers analysed and their Area Under the Curve (AUC) for discriminating bipolar disorder from controls, schizophrenia from controls, and bipolar disorder from schizophrenia.

Biomarkers	BD N=98	SZ N=58	CT N=123	*p*	AUC BD vs. CT	AUC SZ vs. CT	AUC BP vs. SZ
**Blood-based Biomarkers**							
Continuous Biomarkers*							
C-Reactive protein (CRP)	4.56 ± 6.66	4.41 ± 2.94	4.96 ± 7.73	0.845	0.67	0.50	0.65
**Immunoglobulin G (IgG)**	10.13 ± 2.05	12.05 ± 3.44	12.16 ± 2.49	**<0.001**	**0.74**	0.53	0.67
**Immunoglobulin G Subclass 1 (IgG1)**	5.18 ± 1.38	6.79 ± 2.31	6.36 ± 1.74	**<0.001**	0.70	0.54	**0.72**
**Immunoglobulin G Subclass 2 (IgG2)**	3.65 ± 1.48	3.70 ± 1.28	4.21 ± 1.41	**0.007**	**0.62**	0.60	0.53
Immunoglobulin G Subclass 3 (IgG3)	686.39 ± 375.38	701.47 ± 319.99	743.79 ± 365.78	0.477	0.55	0.52	0.53
Immunoglobulin G Subclass 4 (IgG4)	339.54 ± 320.72	441.77 ± 330.09	383.09 ± 350.03	0.185	0.53	0.57	0.61
Immunoglobulin M (IgM)	1.03 ± 0.44	0.97 ± 0.43	1.53 ± 4.45	0.338	0.54	0.57	0.53
Categorical Biomarkers#							
Anti-nuclear factor (ANF)	45.92 (45/98)	29.31 (17/58)	42.28 (52/123)	0.107	0.50	0.57	**0.58**
Antibodies anti-DNA G (anti-DNA G)	8.16 (8/98)	3.45 (2/58)	3.25 (4/123)	0.172	0.53	0.51	0.52
Antibodies anti-DNA M (anti-DNA M)	2.04 (2/98)	1.72 (1/58)	3.25 (4/123)	0.821	0.51	0.50	0.51
Soluble extractable nuclear antigens antibodies (anti-ENA)	1.02 (1/98)	1.72 (1/58)	1.63 (2/123)	0.921	0.50	0.51	0.50
Anti-neutrophil cytoplasmic antibodies (ANCA)	9.18 (9/98)	5.17 (3/58)	6.50 (8/123)	0.744	0.51	0.50	0.51
**Anti-cardiolipin antibodies A (ACA A)**	3.06 (3/98)	10.34 (6/58)	17.89 (22/123)	**0.001**	**0.57**	**0.54**	**0.54**
Anti-cardiolipin antibodies AM (ACA M)	3.06 (3/98)	3.45 (2/58)	8.13 (10/123)	0.193	0.53	0.52	0.50
Beta-2 Glycoprotein 1 Antibodies G (anti-beta2gp1G)	1.02 (1/98)	0.00 (0/58)	0.00 (0/123)	0.350	0.51	0.50	0.51
Beta-2 Glycoprotein 1 Antibodies M (anti-beta2gp1M)	3.06 (3/98)	0.00 (0/58)	3.25 (4/123)	0.191	0.50	0.52	0.52
Rheumatoid factor (RF)	19.39 (19/98)	25.86 (15/58)	30.08 (37/123)	0.176	0.55	0.51	0.54
Anti-citrullinated protein antibodies (anti-CCP)	7.14 (7/98)	18.97 (11/58)	6.50 (8/123)	0.071	0.51	0.55	0.56
Anti-myelin antibodies (anti-Myelin)	0.00 (0/98)	1.72 (1/58)	0.00 (0/123)	0.344	0.52	0.50	0.53
Anti-myelin associated glycoprotein antibodies (anti-MAG)	1.02 (1/98)	0.00 (0/58)	1.63 (2/123)	0.385	0.53	0.51	0.51
Anti-tissue transglutaminase G (anti-TTGG)	1.02 (1/98)	3.45 (2/58)	1.63 (2/123)	0.081	0.53	0.51	0.52
**Antibodies anti-Gliadin A (anti-Gliadin A)**	3.06 (3/98)	10.34 (6/58)	3.25 (4/123)	**0.034**	0.51	**0.54**	**0.55**
Herpes simples virus 1 (HSV1)	64.29 (63/98)	56.90 (33/58)	68.29 (84/123)	0.331	0.52	0.56	0.54
**Herpes simples virus 2 (HSV2)**	23.47 (23/98)	8.62 (5/58)	26.83 (33/123)	**0.010**	0.52	**0.59**	0.57
Cytomegalovirus (CMV)	58.16 (57/98)	55.17 (32/58)	67.48 (83/123)	0.190	0.55	0.56	0.51
**Toxoplasma Gondii IgG**	79.59 (78/98)	63.79 (37/58)	56.10 (69/123)	**0.001**	**0.62**	0.54	**0.58**
